# Teng-Long-Bu-Zhong-Tang, a Chinese herbal formula, enhances anticancer effects of 5 - Fluorouracil in CT26 colon carcinoma

**DOI:** 10.1186/1472-6882-13-128

**Published:** 2013-06-08

**Authors:** Shan Deng, Bing Hu, Hong-Mei An, Qin Du, Ling Xu, Ke-Ping Shen, Xiu-Feng Shi, Meng-Meng Wei, Yang Wu

**Affiliations:** 1 Department of Oncology, Longhua Hospital, Shanghai University of Traditional Chinese Medicine, Shanghai 200032, PR China; 2 Institute of Traditional Chinese Medicine in Oncology, Longhua Hospital, Shanghai University of Traditional Chinese Medicine, Shanghai 200032, PR China; 3 Department of Science & Technology, Longhua Hospital, Shanghai University of Traditional Chinese Medicine, Shanghai 200032, PR China; 4 Department of Pharmacy, Longhua Hospital, Shanghai University of Traditional Chinese Medicine, Shanghai 200032, PR China; 5 State Key Laboratory of Biotherapy and Cancer Center, West China Hospital, West China Medical School, Sichuan University, Keyuan Fourth Road, Chengdu, Sichuan 610072, PR China

**Keywords:** Colon carcinoma, Chinese herbal formula, Apoptosis, Cell senescence, Angiogenesis

## Abstract

**Background:**

Colorectal cancer remains one of the leading causes of cancer death worldwide. Traditional Chinese Medicine (TCM) has played a positive role in colorectal cancer treatment. There is a great need to establish effective herbal formula for colorectal cancer treatment. Based on TCM principles and clinical practices, we have established an eight herbs composed formula for colorectal cancer treatment, which is Teng-Long-Bu-Zhong-Tang (TLBZT). We have demonstrated the anticancer effects of TLBZT against colorectal carcinoma *in vitro*. In present study, we evaluated the anticancer potential of TLBZT, used alone or in combination with low dose of 5-Fluorouracil (5-Fu), in CT26 colon carcinoma *in vivo*.

**Methods:**

CT26 colon carcinoma was established in BALB/c mice and treated with TLBZT, 5-Fu, or TLBZT plus 5-Fu. The tumor volumes were observed. Apoptosis was detected by TUNEL assay. Caspases activities were detected by colorimetric assay. Cell senescence was indentified by senescence β-galactosidase staining. Gene expression and angiogenesis was observed by immunohistochemistry or western blot.

**Results:**

TLBZT significantly inhibited CT26 colon carcinoma growth. TLBZT elicited apoptosis in CT26 colon carcinoma, accompanied by Caspase-3, 8, and 9 activation and PARP cleavage, and downregulation of XIAP and Survivin. TLBZT also induced cell senescence in CT26 colon carcinoma, with concomitant upregulation of p16 and p21 and downregulation of RB phosphorylation. In addition, angiogenesis and VEGF expression in CT26 colon carcinoma was significantly inhibited by TLBZT treatment. Furthermore, TLBZT significantly enhanced anticancer effects of 5-Fu in CT26 colon carcinoma.

**Conclusions:**

TLBZT exhibited significantly anticancer effect, and enhanced the effects of 5-Fu in CT26 colon carcinoma, which may correlate with induction of apoptosis and cell senescence, and angiogenesis inhibition. The present study provides new insight into TCM approaches for colon cancer treatment that are worth of further study.

## Background

Colorectal cancer is the third most commonly diagnosed cancer in males and the second in females worldwide. The incidence of colorectal cancer is increasing due to smoking, lack physical activities, overweight and obesity, red and processed meat consumption, and excessive alcohol consumption [1]. The current treatment of colorectal cancer mainly depends on surgery, chemotherapy, radiotherapy and targeted therapy. However, the curative effect of these treatments are less than satisfactory, the 5-year overall survival after resection for colon cancer is about 60% [2], the 5-year survival for metastatic colorectal cancer is only approximately 10% [3]. Colorectal cancer remains the fourth leading cause of cancer death in men and the third in women worldwide [1]. Clearly, development of novel approach for colorectal cancer treatment is highly warranted.

In China, Traditional Chinese Medicine (TCM) has played a positive role in colorectal cancer treatment. TCM has been confirmed to effectively enhance curative effects and reduce toxic side effects of chemotherapy, palliate clinical syndrome, prevent recurrence and metastasis, improve quality of life and immune function, and prolong survival time in colorectal cancer [4]. The personalized TCM therapy is Syndrome Based Differential Treatment. In Chinese herbalism, every herb has its own characteristics. Diseases can be effectively treated by combining herbs based on their various features. Combinations of multiple herbs guided by TCM theories, called Chinese herbal formula, are the major application form of Chinese herb. Due to the lack of appropriate ancient Chinese herbal formula for cancer, most TCM physicians combine multiple herbs for a formula or prescription based on the patient’s illness and body condition, TCM principles, pharmacological studies and personal experience [5]. There is a great need to establish effective herbal formula for colorectal cancer treatment.

According to the TCM theories and clinical observations, the pathogenesis of colorectal cancer is related to damp-heat, toxicity accumulation, and spleen-deficiency [4,6,7]. Based on the therapeutic method of clearing heat-toxicity, eliminating dampness and tonifying Pi (spleen), and the modern principle of anticancer and anti-angiogenesis, and TCM clinical practices, we have established an eight herbs composed formula for colorectal cancer treatment, which is Teng-Long-Bu-Zhong-Tang (TLBZT). We have demonstrated TLBZT may inhibit proliferation, activate Caspases to induce apoptosis, upregulate p16 and p21 and downregulate RB phosphorylation to induce cell senescence in colon carcinoma cells *in vitro*[8,9]. In present study, we evaluated the anticancer effects of TLBZT, used alone and in combination with low dose of 5-Fluorouracil (5-Fu), in CT26 colon carcinoma *in vivo*.

## Methods

### Materials

DMEM medium and fetal bovine serum was obtained from Hyclone (Logan, UT). 5-Fu injection was bought form Xudong Haipu Pharmaceutical Co., Ltd (Shanghai, China). FragEL^™^ DNA Fragmentation Detection Kit was purchased from EMD Millipore (Darmstadt, Germany). Senescence β-Galactosidase Staining Kit and PARP antibody were from Cell Signaling Technology (Danvers, MA). Caspase-3, Caspase-8 and Caspase-9 Activity Assay Kit were obtained from Beyotime Institute of Biotechnology (Jiangshu, China). Antibody against p21 was purchases from Boster Bio-engineering Limited Company (WuHan, China). XIAP, Survivin, GAPDH and pRB antibodies were purchased from Bioworld Technology (St. Louis Park, MN). Antibody against p16 was purchased from Proteintech (Chicago, IL). Antibodies against CD31 and VEGF were the product of from Santa Cruz Biotechnology (Santa Cruz, CA).

### Preparation of TLBZT

The herbs used in TLBZT formula (Chinese patent ZL200910197565.2) are the roots of *Actinidia chinensis* (Teng-Li-Geng) 30 g, *Solanum nigrum* (Long-Kui) 15 g, *Duchesnea indica* (She-Mei) 15 g, *Atractylodes macrocephala Koidz* (Bai-Zhu) 9 g, *Poria cocos* (Fu-Ling) 15 g, *Coix* seed (Yi-Yi-Ren) 30 g, *Mistletoe* (Hu-Ji-Sheng) 15 g, and *Scutellaria barbata* (Ban-Zhi-Lian) 30 g. All those herbs were from the herb store in Longhua Hospital according to the original proportion, and decocted twice with 8-fold volume of distilled water for 1 hour. The decoction were collected, filtered, merged and concentrated to 1.5 g/mL (equivalent to crude herb materials), and stored at 4°C.

For Gas chromatography–mass spectrometry (GC/MS) analysis, TLBZT were further extracted with dichloromethane and diethyl ether, and passed through 0.22 μm filter. GC/MS analysis of TLBZT extract was performed by GCMS6800 (Skyray Instrument, Jiangsu, China) equipped with a DB-5ms column (30 m × 0.25 mm × 0.25 μm) (Agilent technologies, CA). Helium was used as carrier gas at a constant flow rate of 1 mL/min. An injection volume of 1 μL was employed in splitless mode. Injector and ion-source were maintained at 280°C and 230°C, respectively. The mass-scan range was 50–500. The GC/MS profile of TLBZT is presented in Additional file 1: Figure S1.

### Cell culture and animal model

Murine colon carcinoma CT26 cells were obtained from obtained from Cell Bank of Type Culture Collection of Chinese Academy of Sciences. CT26 cells were grown in DMEM medium with 10% FBS, penicillin (100 U/mL) and streptomycin (100 μg/mL) and maintained at 37°C with 5% CO_2_ in a humidified atmosphere. Female (6–7 weeks old) BALB/c mice (obtained from Shanghai SLAC Laboratory animal center) were acclimated for one week and were fed with animal chow and water ad libitum in SPF animal laboratory of Longhua Hospital.

The mice were injected s.c. with 1 × 10^6^ CT26 cells in 100 μl PBS in the right flank. When the tumors were palpable, the mice were randomly divided into 4 groups (n=10 mice/group), and intragastric administered with TLBZT (22.5 g/kg/0.3 ml, equivalent to crude herb materials, once a day) or same volume of distilled water, or i.p. administered with 5-FU (30 mg/kg/0.3 ml, once a week), or treated with both TLBZT and 5-Fu. Tumor width (W) and length (L) were measured every 3 days by calipers. The tumor volume (Tv) was calculated according to the formula: Tv = 0.52 × L × W^2^. After three weeks of treatment, the mice were sacrificed, and the tumors were removed, weighed and subjected to further experiments. All studies involving mice were approved by the Longhua Hospital Animal Care and Use Committee.

### TUNEL assay

Apoptotic cells were identified by TUNEL (terminal deoxynucleotidyl transferase-mediated nick end labeling) assay following the manufacturer’s guide. Images were captured by the Olympus microscope at ×200 magnification. The apoptotic cells were counted by Image-Pro Plus 6.0 software.

### Caspases activities assay

The activities of Caspases were detected by Caspase-3, 8 and 9 Activity Assay Kit. According to the manufacturer's protocol, the tumor samples were homogenized, and the supernatant were collected and determined protein concentration. Then, the supernatant were respectively incubated with Ac-DEVD-pNA (Caspase-3), Ac-IETD-pNA (Caspase-8) and Ac-LEHD-pNA (Caspase-9) in assay buffer at 37°C for 2 hours. Finally, the production of p-nitroaniline was monitored by microplate reader at wavelength of 405 nm.

### Senescence β-galactosidase staining

Senescent cells in tumor samples were identified by Senescence β-galactosidase (SA-β-gal) staining was performed according to the manufacturer's protocol. Images were captured by Olympus microscope at ×200 magnification and analyzed by Image-Pro Plus 6.0 software.

### Immunohistochemistry

The paraffin-embedded tumor tissues were sectioned (5 μm), deparaffinized, blocked with 3% hydrogen peroxide and washed with PBS. For immunostaining, sections were probed with antibodies against cleaved PARP (1:100), XIAP (1:200), Survivin (1:200), p21 (1:200), p16 (1:200), pRB (1:200), CD31 (1:100), and VEGF (1:100) at 4°C overnight, followed by incubation with secondary antibody and visualized using 3,3-diaminobenzidine as chromagen. Sections were counterstained with hematoxylin and mounted with glass coverslips. Images were captured by the Olympus microscope, and analyzed by Image-Pro Plus 6.0 software.

### Western blot

Western blots were performed as described previously [9]. Briefly, after three weeks treatment, CT26 carcinomas (3 tumors/group) were collected, lysed, combined and subjected to 8–10% SDS-PAGE gel, and transferred onto a nitrocellulose membrane (Amersham). The transferred membrane were blocked with 5% non-fat milk, washed, and probed with antibodies against cleaved PARP (1:1000), XIAP (1:1000), Survivin (1:1000), p16 (1:1000), p21 (1:1000), pRB (1:1000), VEGF (1:500) or GAPDH (1:2000). Blots were then washed and incubated with IRDye 700- conjugated (1:3000) or IRDye 800-conjugated (1:5000) secondary antibodies (Rockland Immunochemicals), and visualized in Odyssey Infrared Imaging System (LI-COR Biosciences).

### Data analysis

Results were expressed as mean ± standard deviation, and the differences between groups were compared by one-way ANOVA. Differences were considered significant at *P*<0.05.

## Results

### TLBZT and 5-Fu inhibited CT26 colon carcinoma growth

**Figure 1:**
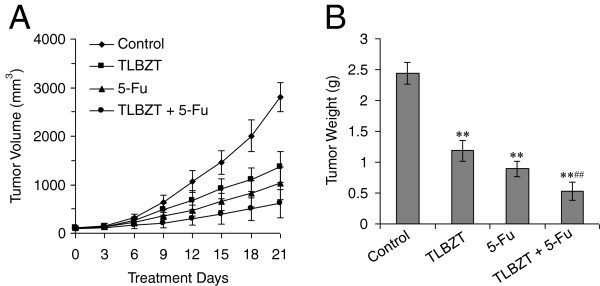
**TLBZT and 5-FU inhibited CT26 carcinoma growth.** Female BALB/c mice were injected s.c. with 1 × 10^6^ CT26 cells. When the tumors were palpable, the mice were randomized to receive treatment with TLBZT, 5-Fu, TLBZT plus 5-Fu, or distilled water as a control. Tumor volumes were monitored every three days (**A**). After three weeks of treatment, the tumors were removed and weighed (**B**). **P<0.01, versus control group, ^##^*P*<0.01, versus TLBZT or 5-Fu group.

To observe the effect of TLBZT on tumor growth, CT26 colon carcinoma was established in BALB/c mice. When the tumors were palpable, the mice were treated with TLBZT, 5-Fu, TLBZT plus 5-Fu, or distilled water. As shown in Figure 1, tumors grew progressively in control group. TLBZT or 5-FU significantly inhibited CT26 colon carcinoma growth as demonstrated by tumor volume and tumor weight (*P* < 0.01). TLBZT combined with 5-Fu significantly increased the effects in inhibiting tumor growth than either treatment alone (*P* < 0.01).

### TLBZT and 5-Fu induced apoptosis in CT26 colon carcinoma

**Figure 2:**
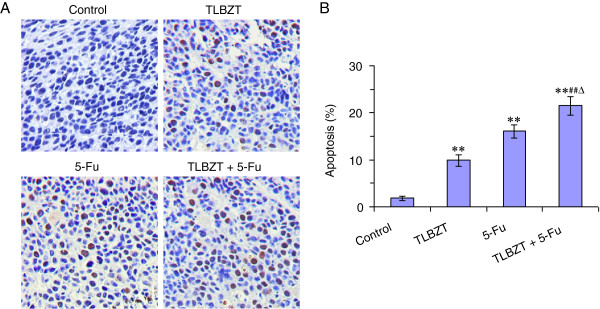
**TLBZT and 5-FU induced apoptosis in CT26 carcinoma.** After three weeks treatment, CT26 carcinomas were collected and subjected to TUNEL assay (3 tumors/group) and observed under microscope (×200) (**A**). The apoptotic cells were counted in 3 high power fields (HPF) in each slide. The percentage of apoptosis cells were expressed as means ± SD (**B**). **P<0.01, versus control group. ^##^*P*<0.01, versus TLBZT group, ^Δ^*P*<0.05, versus 5-Fu group.

After three weeks of treatment, the tumor were collected and embedded with paraffin. The apoptotic tumor cells were determined by the TUNEL assay. As shown in Figure 2, TUNEL positive cells were represented brown staining, the TUNEL positive cells were significantly increased in TLBZT and 5-Fu group and compared with controls (*P*<0.01). The combination group showed more apoptotic cells than TLBZT or 5-Fu alone (*P*<0.05).

### TLBZT and 5-Fu activated Caspases

**Figure 3:**
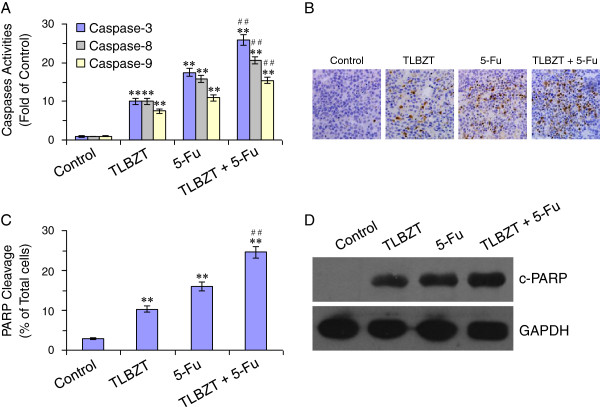
**TLBZT and 5-FU activitied Caspases.** After three weeks treatment, CT26 carcinomas (3 tumors/group) were removed, homogenized, and subjected to Caspase-3, Caspase-8 and Caspase-9 activities assay by cleavage of specific substrate. Caspases activities were expressed as fold activation over control (**A**). Cleavage of PARP was detected by immunohistochemistry (3 tumors/group) and observed under microscope (×200) (**B**). Cleaved PARP positive cells (3 HPF/slide) were counted by Image-Pro Plus 6.0 software and expressed as percentage of total cells (**C**). **D**, PARP Cleavage in CT26 carcinomas were further verified with western blot by antibody against cleavage PARP (c-PARP). GAPDH was used as a loading control. **P<0.01, versus control group, ^##^*P*<0.01, versus TLBZT or 5-Fu group.

Cell apoptosis is executed by a Caspase cascade [10], so we further tested Caspase-3, 8 and 9 activities after drug treatment. As shown in Figure 3A, after three weeks of treatment, Caspase-3, 8 and 9 were significantly activated in TLBZT and 5-Fu group and compared with controls (*P*<0.01). Combinational treatment with TLBZT and 5-Fu was showed more effective in Caspase-3, 8 and 9 activation than TLBZT or 5-Fu treatment alone (*P*<0.01). In addition, PARP, one of the earliest substrates of Caspase-3 during apoptosis [11], was cleavaged after TLBZT or 5-Fu treatment (Figure 3B and C). Cleavaged PRAP was significantly increased in TLBZT plus 5-Fu group than TLBZT or 5-Fu group (*P*<0.01). We also confirmed PARP cleavage by western blot, the results of western blot were consistent with immunohistochemistry (Figure 3D).

### Effects of TLBZT and 5-Fu on XIAP and Survivin expression

**Figure 4:**
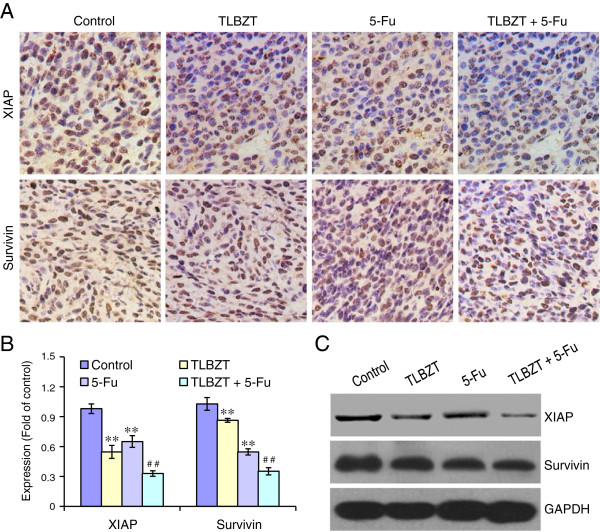
**Effects of TLBZT and 5-FU on XIAP and Survivin expression.** After three weeks treatment, expression of XIAP and Survivin in CT26 carcinoma (3 tumors/group) were detected by immunohistochemistry and observed under microscope (×200) (**A**). The mean optical density (MOD) of XIAP and Survivin (3 HPF/slide) were analyzed by Image-Pro Plus 6.0 software, and expressed as fold of control (**B**). **C**, expression of XIAP and Survivin in CT26 carcinomas were further verified with western blot by specific antibody. GAPDH was used as a loading control. **P<0.01, versus control group, ^##^*P*<0.01, versus TLBZT or 5-Fu group.

It has been reported inhibitor of apoptosis proteins, such as XIAP and Survivin are overexpressed in colorectal cancer [12,13]. We also observed XIAP and Survivin expression in CT26 colon carcinoma after three weeks of drug treatment. As shown in Figure 4, XIAP and Survivin were overexpressed in CT26 colon carcinoma. TLBZT or 5-Fu treatment significantly inhibited XIAP and Survivin expression and compare with controls (*P*<0.01). TLBZT combined with 5-Fu significantly increased the inhibitory effects on XIAP and Survivin expression than either treatment alone (*P*<0.01).

### TLBZT induced cell senescence in CT26 colon carcinoma

**Figure 5:**
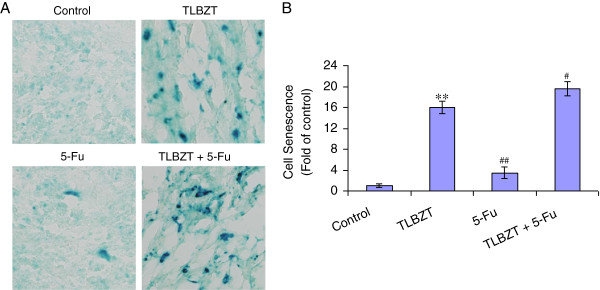
**Effects of TLBZT and 5-FU on cell senescence in CT26 carcinoma. A**, after three weeks treatment, CT26 carcinomas (3 tumors/group) were removed and frozen cut into 7μm sections, fixed, reacted with SA-β-gal staining solution overnight and observed under microscope (×200). **B**, SA-β-gal positive staining was counted in 3 HPF in each slide and expressed as fold over control. **P<0.01, versus control group, ^##^*P*<0.01, versus TLBZT group, ^#^*P*<0.05, versus TLBZT group.

We have demonstrated TLBZT may induce cell senescence in colon carcinoma cells *in vitro*[9], so we further detected cell senescence in CT26 colon carcinoma after three weeks of treatment. The senescent cells were identified by SA-β-gal staining at an acidic pH as a marker [14], and showed blue staining. TLBZT treatment resulted in significant cell senescence in CT26 colon carcinoma compared with controls (*P*<0.01). To our surprise, cell senescence in 5-Fu treated CT26 colon carcinoma was few compared with TLBZT (*P*<0.01) (Figure 5).

### Effects of TLBZT cell senescence related gene expression

**Figure 6:**
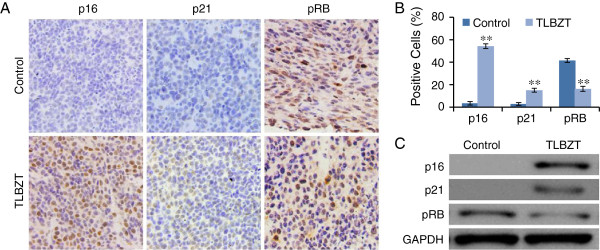
**Effects of TLBZT on cell senescence related gene expression.** After three weeks treatment, p16 and p21, and RB phosphorylation in CT26 carcinomas (3 tumors/group) were detected by immunohistochemistry and observed under microscope (×200) (**A**). The p21, p16 and RB phosphorylation positive cells (3 HPF/slide) were counted by Image-Pro Plus 6.0 software, and expressed as mean ± SD (**B**). **C**, p16 and p21 expression and RB phosphorylation was further detected with western blot by specific antibody. GAPDH was used as a loading control. **P<0.01, versus control group.

It has been demonstrated p21, p16 and RB phosphorylation plays a central role in cell senecescence [15]. We examined p16, p21 and RB phosphorylation in CT26 colon carcinoma after three weeks of TLBZT treatment by immunohistochemistry and western blot. As shown in Figure 6, TLBZT significantly upregulated p16 and p21 expression, and downregulated RB phosphorylation in CT26 colon carcinoma and compared with controls (*P*<0.01).

### TLBZT inhibited angiogenesis and VEGF expression

**Figure 7:**
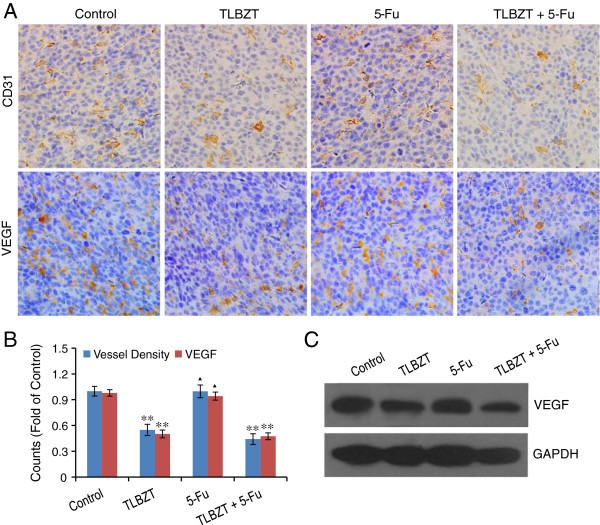
**Effects of TLBZT and 5-FU on angiogenesis. A**, after three weeks treatment, angiogenesis and VEGF expression in CT26 carcinomas (3 tumors/group) were detected by immunohistochemistry and observed under microscope (×200). **B**, vessel density was determined via counting the number of the microvessels per high-power field (3 HPF/slide), and the MOD of VEGF (3 HPF/slide) were analyzed by Image-Pro Plus 6.0 software, and expressed as fold over control. **C**, VEGF expression in CT26 carcinomas were further verified by western blot. GAPDH was used as a loading control. **P<0.01, versus control group. ^▲^*P*>0.05, versus control group.

Some herbs in TLBZT, such as *Scutellaria barbata* and *Mistletoe* have been reported to possess anti-angiogenesis potential [16-19]. We suppose that the reduction of tumor growth by TLBZT treatment may be partially involved in the inhibition of angiogenesis. Angiogenesis within CT26 colon carcinoma tissue was estimated by immunohistochemistry with an antibody reactive to CD31 as an endothelial marker [20,21]. The result showed TLBZT treatment resulted in apparent inhibition of angiogenesis in CT26 colon carcinoma compared with control groups (*P*<0.01). In addition, expression of VEGF was also significantly inhibited by TLBZT treatment compared with control group (*P*<0.01). (Figure 7).

## Discussion

In TCM, the principle of combining herbs for a Chinese herbal formula is monarch, minister, assistant and guide (Jun, Chen, Zuo and Shi) [5,22]. The monarch herb(s) are the key ingredient in the formula to target the primary cause or syndrome. Minister herb(s) are used to strength the effect of the Monarch herb(s) or address the secondary syndromes. Assistant herb(s) are utilized to reinforce the curative effect of the Monarch or Minister herb(s), or allay the drastic and toxic effect of the Monarch or Minister herb(s). While the Guide herb(s) are applied to harmonize and integrate the effects of other herbs, or direct the formula to act on the target meridian or the offending part of body. However, Guide herb(s) are not indispensable for a formula, based on the specific conditions, Guide herb(s) might be used or not used in a formula.

According to the TCM theories, *Actinidia chinensis, Solanum nigrum* and *Duchesnea indica* are used to against the pathogenic factors of damp-heat and toxicity accumulation, and served as Monarch herbs in TLBZT. In addition to traditional herbal efficacy, *Actinidia chinensis*, *Solanum nigrum* and *Duchesnea indica* also have been proved anticancer potential. It has been reported ethanol extracts from *Actinidia chinensis* may inhibit colon carcinoma LoVo cells and HT-29 cells proliferation, and induce apoptosis in LoVo cells accompanied by Bcl-2/Bax downregulation and Caspase-3 upregulation [23,24]. Components of *Solanum nigrum*, such as solamargine, Solanine, polysaccharide and polyphenol-rich extract of *Solanum nigrum* have demonstrated anticancer effects against various cancer cells [25-28]. Phenolic extract of *Duchesnea indica* can inhibit cervical and ovarian cancer growth through induction of apoptosis and cell cycle arrest [29,30].

*Atractylodes macrocephala Koidz, Poria cocos* and *Coix* seed are used as Minister herbs to target spleen-deficiency, damp and the loss of appetite induced by long-term use of Monarch herbs. In addition to traditional efficacy of tonifying Pi (spleen), *Atractylodes macrocephala Koidz*, *Poria cocos* and *Coix* seed or their components also have been showed anticancer effects against cancer cells. *Atractylodes macrocephala Koidz* extract may inhibit S180 tumor growth [31]. Poricotriol A from *Poria cocos* may induce apoptosis in leukemia HL-60 cells and lung cancer A549 cells [32]. Pachymic acid from *Poria cocos* may reduce cell proliferation and induced apoptosis through mitochondria dysfunction in prostate cancer cells [33]. Kanglaite injection, a *Coix* seed extract, has been widely used as anticancer drug in Chinese oncological clinical [34]. In addition, a variety of compounds from *Coix* seed bran ethanolic extract, such as coixspirolactam D, coixspirolactam E, coixspiroenone, coixspirolactam A, coixspirolactam C, coixlactam, and ficusal, may significantly inhibite breast cancer cells proliferation [35].

*Scutellaria barbata* and *Mistletoe* are Assistant herbs to enhance the effects of Monarch and Minister herbs by their anti-angiogenesis and anticancer effects. It has been reported *Scutellaria barbata* may inhibit angiogenesis in vitro and in colorectal cancer model via suppression of Hedgehog pathway and VEGF [16,17]. *Scutellaria barbata* extract also have been showed cytotoxity effects against human colon cancer cells [36]. Viscum album (*Mistletoe*) may induce apoptosis in endothelial cells and inhibit angiogenesis [18,19]. In addition, *Mistletoe* lectins could inhibit proliferation and induce apoptosis in colon cancer HT-29 cells [37]. TLBZT is an herbal formula fitted with both TCM theories and the principle of anticancer. In present study, we observed TLBZT, alone or in combination with 5-Fu, significantly inhibited CT26 colon carcinoma growth accompanied by apoptosis.

Apoptosis is an evolutionarily conserved cell suicide process that acts to balance mitosis in the development and maintenance of tissue homeostasis for the removal of superfluous, transformed or damaged cells, and has been recognized as a popular target for anticancer therapy [10,38,39]. Two major pathways have been identified in the process of apoptosis. In extrinsic death receptor pathway, the death ligands (FasL, TRAIL, etc.) binds to the death receptors (FAS, TRAIL-R, etc.) which recruits adaptor proteins, such as Fas-associated death domain (FADD), to form ligand-receptor-adaptor protein complex (known as the death-inducing signalling complex, DISC), and then activists Caspase-8, followed by Caspase-3 activation and apoptosis. The intrinsic pathway involves the signals to mitochondria which lead to release of cytochrome C from mitochondria. Released Cytochrome C combines Apaf-1 and Caspase-9 to form apoptosome and activates Caspase-9 which in turn activates Caspases-3, causing the cell to undergo apoptosis. As the members of inhibitor of apoptosis proteins (IAPs), XIAP and Survivin are overexpressed in colorectal cancer, and have been recognized as diagnostic markers and therapeutic targets [12,13,40]. XIAP and Survivin may inhibit activation of Caspases, downregulation of XIAP and Survivin could sensitize colorectal cancer cell to drug induced apoptosis [41,42]. In present study, TLBZT alone or in combination with 5-Fu, significantly induced apoptosis in CT26 colon carcinoma, accompanied by Casapse-3, 8 and 9 activation, and downregulation of XIAP and Survivin, suggested casapses activation and downregulation of XIAP and Survivin may contribute to TLBZT and 5-Fu induced apoptosis.

In addition to apoptosis, cell senescence also contributes to cancer therapeutic response, and has been suggested as a cancer treatment target [43-45]. Cell senescence is a state of stable irreversible cell cycle arrest and loss of proliferative capacity. Senescent cell maintains some metabolic activity but no longer proliferates, and exhibits increased SA-β-gal activity at an acidic pH. Positive of SA-β-gal staining at an acidic pH has been identified as biomarker of cell senescence since 1995 [14]. Cell senescence is closely related to the activation of the CDKN2a (p16^INK4A^)/pRB or CDKN1a (p21^WAF-1/Cip1^)/pRB signaling pathway [15,43-45]. The CDK4 and CDK6 inhibitor p16 participates in regulation of RB phosphorylation, induces cell cycle arrest, and contributes to the induction of cell senescence. p21, an important cell cycle regulator, inhibits a variety of cyclin/CDK complexes, resulted in hypophosphorylation or dephosphorylation of RB protein which binds to E2F and prevents it from activating target genes that are essential in the cell cycle, usually leading to cell cycle arrest. It have been reported natural products, such as Ganoderiol F, *Antrodia camphorata* extract, Liver-Yin tonifying herbs can inhibit cancer cell growth via cell senescence [46-48]. In present study, TLBZT significantly increased SA-β-gal activity accompanied by an increase in p16 and p21, and downregulation of RB phosphorylation, suggested that TLBZT may induce cell senescence in CT26 carcinoma and related to upregulation of p16 and p21 and downregulation of RB phosphorylation.

Angiogenesis, the process of new blood vessel generate from existing vessels, plays a crucial role in tumor growth and metastasis. Angiogenesis has been recognized as an impotent therapeutic target for cancer treatment since it first proposed by Judah Folkman in 1971 [49]. Currently, angiogenesis targeted drugs, such as bevacizumab (monoclonal antibody against VEGF), sorafenib, sunitinib, pazopanib and everolimus have been wildly used in clinical. CD31 or platelet/endothelial cell adhesion molecule-1(PECAM-1) is a widely used marker protein for angiogenesis [20,21]. VEGF, secreted by cancer cells, vascular endothelial cells or tumor associate macrophages, is a major driver of tumor angiogenesis [50,51]. By stimulating vascular endothelial cells proliferation, VEGF can trigger angiogenesis and promote tumor growth. In present study, we detected TLBZT significantly inhibited angiogenesis in CT26 colon carcinoma with concomitant downregulation of VEGF, suggested that anti-angiogenesis may contribute to TLBZT mediated anticancer effects. In TLBZT, *Actinidia chinensis*[52], *Solanum nigrum*[53], *Duchesnea indica*[54], *Scutellaria barbata*[16,17], and *Mistletoe*[18,19] or their ingredients have been demonstrated anti-angiogenesis effects. The components and the precise mechanism responsible for TLBZT induced anti-angiogenesis effects need to be further explored.

## Conclusion

Our study demonstrated that TLBZT exhibited significantly anticancer effect, and enhanced the effects of 5-Fu in CT26 colon carcinoma, which may correlate with induction of apoptosis and cell senescence, and angiogenesis inhibition. The present study provides new insight into TCM approaches for colon cancer treatment that are worth of further study.

## Competing interests

The authors declare that they have no competing interests.

## Authors’ contributions

DS performed the study and drafted the manuscript. HB designed the study and revised the manuscript. HB and AHM established the herbal formula. DQ, XL, SKP and WMM participated the study. SXF prepared the herbs. WY helped data analysis. All authors read and approved the final manuscript.

## Pre-publication history

The pre-publication history for this paper can be accessed here:

http://www.biomedcentral.com/1472-6882/13/128/prepub

## Supplementary Material

Additional file 1: Figure S1 GC/MS profile of TLBZT. Click here for file
