# Supplementary figures and images for: Teng-Long-Bu-Zhong-Tang, a Chinese herbal formula, enhances anticancer effects of 5 - Fluorouracil in CT26 colon carcinoma
Source: BMC Complement Altern Med. 2013 Jun 8;13:128. doi: 10.1186/1472-6882-13-128 (PMC3702481; doi:10.1186/1472-6882-13-128)

## Slide 1
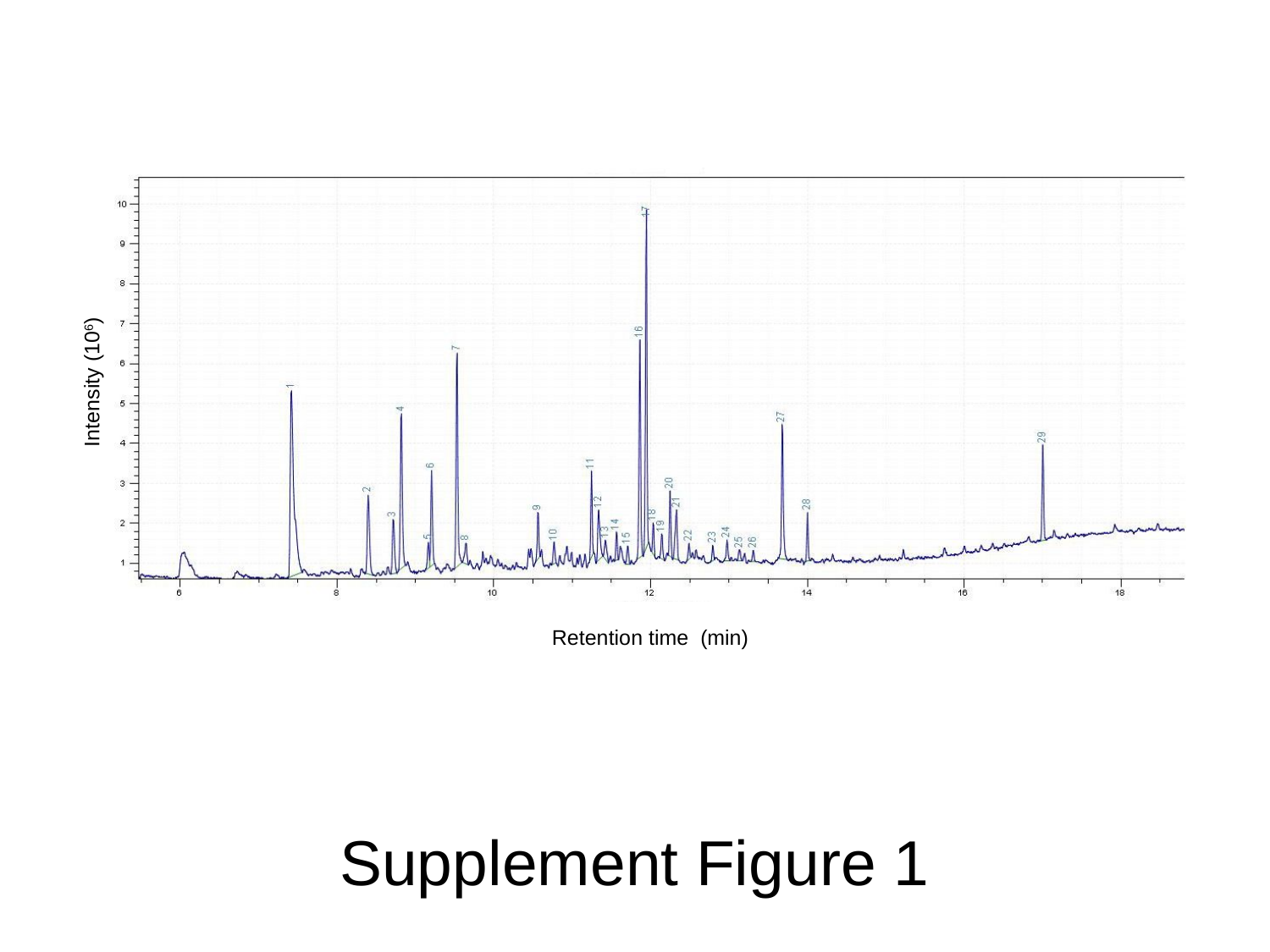

Intensity (106)
Retention time (min)
Supplement Figure 1

Supplement: Additional file 1: Figure S1 — GC/MS profile of TLBZT. [file 1472-6882-13-128-S1.ppt]
